# Using Bayesian inference to estimate plausible muscle forces in musculoskeletal models

**DOI:** 10.1186/s12984-022-01008-4

**Published:** 2022-03-23

**Authors:** Russell T. Johnson, Daniel Lakeland, James M. Finley

**Affiliations:** 1 Division of Biokinesiology and Physical Therapy, University of Southern California, Los Angeles, CA USA; 2 Lakeland Applied Sciences, Los Angeles, CA USA; 3 Department of Biomedical Engineering, University of Southern California, Los Angeles, CA USA; 4 Neuroscience Graduate Program, University of Southern California, Los Angeles, CA USA

**Keywords:** Sensitivity analysis, Objective function, Markov Chain Monte Carlo

## Abstract

**Background:**

Musculoskeletal modeling is currently a preferred method for estimating the muscle forces that underlie observed movements. However, these estimates are sensitive to a variety of assumptions and uncertainties, which creates difficulty when trying to interpret the muscle forces from musculoskeletal simulations. Here, we describe an approach that uses Bayesian inference to identify plausible ranges of muscle forces for a simple motion while representing uncertainty in the measurement of the motion and the objective function used to solve the muscle redundancy problem.

**Methods:**

We generated a reference elbow flexion–extension motion and computed a set of reference forces that would produce the motion while minimizing muscle excitations cubed via OpenSim Moco. We then used a Markov Chain Monte Carlo (MCMC) algorithm to sample from a posterior probability distribution of muscle excitations that would result in the reference elbow motion. We constructed a prior over the excitation parameters which down-weighted regions of the parameter space with greater muscle excitations. We used muscle excitations to find the corresponding kinematics using OpenSim, where the error in position and velocity trajectories (likelihood function) was combined with the sum of the cubed muscle excitations integrated over time (prior function) to compute the posterior probability density.

**Results:**

We evaluated the muscle forces that resulted from the set of excitations that were visited in the MCMC chain (seven parallel chains, 500,000 iterations per chain). The estimated muscle forces compared favorably with the reference forces generated with OpenSim Moco, while the elbow angle and velocity from MCMC matched closely with the reference (average RMSE for elbow angle = 2°; and angular velocity = 32°/s). However, our rank plot analyses and potential scale reduction statistics, which we used to evaluate convergence of the algorithm, indicated that the chains did not fully mix.

**Conclusions:**

While the results from this process are a promising step towards characterizing uncertainty in muscle force estimation, the computational time required to search the solution space with, and the lack of MCMC convergence indicates that further developments in MCMC algorithms are necessary for this process to become feasible for larger-scale models.

**Supplementary Information:**

The online version contains supplementary material available at 10.1186/s12984-022-01008-4.

## Background

Movement scientists are often interested in quantifying the timing and magnitude of muscle forces during motions like walking or reaching to understand causal links between muscle mechanics and movement. Accurately and reliably estimating individual muscle forces has implications for how well researchers can evaluate muscle function to help guide surgical interventions, inform the design of prosthetics and orthotics, and estimate other clinically relevant outputs (e.g., joint contact forces) [1–6]. Measuring muscle forces in vivo is difficult to do, except on a limited scale (e.g., triceps surae forces [7]), but most often the methodology is far too invasive to use with human participants. Instead, researchers often use experimental data combined with musculoskeletal modeling to estimate muscle forces during a movement [8–11]. Several methods have been developed to estimate individual muscle forces during a motion, including static optimization, computed muscle control, as well as direct collocation or simulated annealing methods to solve for muscle forces [e.g., [12–15]]. Typically, these methods result in a single force trajectory for each muscle that optimizes a chosen objective function for a given musculoskeletal model and experimental motion. However, accurately solving for muscle forces remains difficult because the musculoskeletal system is redundant (an infinite combination of muscle forces can often give rise to the same joint moment) [16], and simulations of movement depend on experimental data and a variety of parameters that are prone to uncertainties [17, 18].

The uncertainty associated with muscle force estimation can arise from the uncertainty with which we mathematically represent how the central nervous system distributes muscle forces amongst agonist muscles [8, 12], errors in marker placement and skin movement relative to anatomical landmarks [19, 20], and modeling assumptions related to muscle parameters [21, 22]. Typical representations of motor control assume that the central nervous system attempts to minimize some objective function (e.g., minimize muscle fatigue or metabolic energy cost [8, 17, 23]). Choosing an appropriate objective function for a particular motion is difficult because it is not known exactly how the nervous system distributes forces across muscles. In reality, the nervous system is unlikely to generate an “optimal” motion under any hypothesis represented by a simple objective function, as biological systems may find “good-enough” solutions to movement objectives, but these may not necessarily be optimal behavior as defined in trajectory optimization problems [24]. The unknowns associated with choosing an appropriate objective function for musculoskeletal simulations are problematic because muscle force estimates are sensitive to the objective function chosen [17, 23, 25–27]. There are other aspects of musculoskeletal modeling that can lead to uncertainty in muscle force estimation, such as variability in marker placement, movement artifact, and unknown model parameters which can also play a role in impacting the computed muscle forces [18–21, 28–30]. With uncertainties in the motor control model, the measured data, and the musculoskeletal model, the single solutions typically obtained from standard optimizations conceal the inherent uncertainty we have about the predicted muscle forces. By better quantifying the uncertainty in muscle force estimation, researchers can evaluate experimental design approaches capable of reducing uncertainty and better predict whether a designed intervention would lead to meaningful changes in muscle force production.

One approach to quantifying uncertainty in musculoskeletal modeling is to treat the objective function as unknown while keeping the other model parameters fixed. There have been a few different methods developed to try to capture some of the uncertainty associated with choosing an objective function and how it would affect the estimated muscle forces for a given model or motion [31–33]. These approaches have used mathematical mappings between joint torques and muscle activations to compute upper and lower bounds on the muscle forces for each muscle over time. Instead of choosing an explicit objective function to solve the muscle redundancy problem, these methods instead solve for the upper and lower bounds then assume the muscle forces lie somewhere in between. However, these ranges include solutions that would only be possible with extreme co-activation of agonist and antagonist muscles. Extreme co-activation is unlikely to occur in most healthy human sub-maximal movements, especially if muscle forces are distributed in a way that is sensitive to the physiological load (or effort) across individual muscles [23] or reduces metabolic cost. One previous study used EMG data as a way to provide some bounds on the range of possible muscle forces [31], however the remaining muscles without EMG data were left unbounded and therefore still had vast ranges of possible muscle forces. Additionally, there are other limitations to directly using EMG data for this approach, such as uncertainty about how to normalize EMG, resolving forces from EMG, and collecting EMG from deep muscles [34–37]. Therefore, there is a critical gap in the field of musculoskeletal modeling and simulation between (a) solving for muscle forces with an explicit, but uncertain, objective function (or subset of them) and (b) solving for the broad range of possible muscle forces that include muscle force combinations that are not realistic without extreme co-activation of agonist muscle groups.

Bayesian inference methods are well suited for problems where we want to constrain the set of plausible solutions based on prior evidence and knowledge of the musculoskeletal system. This evidence could include information about physiology, measurement errors, and model-based uncertainties. Bayesian inference problems are defined with a prior function (a set of plausible assumptions about the problem), a likelihood function (provided by observed data about a set of parameters), and a posterior function (a quantification of the plausible values of a set of parameters) [38]. The logarithmic forms of these functions (log Prior, log Likelihood, and log Posterior) are preferred for a Bayesian inference problem because it is computationally more stable and effective.

One common way to sample from the solution space is to use a Markov Chain Monte Carlo (MCMC), which is a computer-driven sampling method that allows us to characterize a posterior distribution without knowing all of the distribution’s properties. The MCMC analysis generates the random samples (or proposals) from a multi-dimensional parameter space via a sequential process according to rules that compare consecutive proposals, and this generates a ‘chain’ of proposals [39, 40]. One unique property of a MCMC chain is that new proposals are based on the previous proposal, but do not depend on any proposal prior to the previous one [38]. Then, as the MCMC algorithm is iterated, the set of visited locations is used as a sample from the Bayesian posterior distribution of the unknown parameters [40, 41], which numerically represents a set of equally plausible parameter vectors that could produce a result that is similar to the observed data.

Our aim is to evaluate the feasibility of using Bayesian inference methods to quantify the plausible range of muscle forces for human motion. For this initial feasibility assessment, we developed a prior based on a commonly used objective function (integrated muscle excitation cubed), while keeping other musculoskeletal model parameters constant throughout the study (e.g., peak isometric muscle forces, tendon slack lengths). Our prior was based on physiological hypotheses that muscle forces are distributed amongst agonist muscles and that co-activation of agonist and antagonist muscles is typically low for healthy human motions [23, 42]. We used an MCMC sampling algorithm in MATLAB and simulated an elbow flexion–extension task (reference motion) using OpenSim to explore the plausible excitations that could give rise to the reference joint trajectory. We then compared the excitations from MCMC to the known original simulation that generated the reference motion. Our aim for this paper is to present a workflow for building a Bayesian model and performing MCMC analysis to sample plausible muscle forces for a measured motion with a musculoskeletal model. Ultimately, we hope that this workflow will allow movement scientists to appropriately account for uncertainties in measurement, model structure, model parameters, and assumed cost functions in musculoskeletal simulations.

## Methods

### Musculoskeletal model and generating reference data

**Fig. 1:**
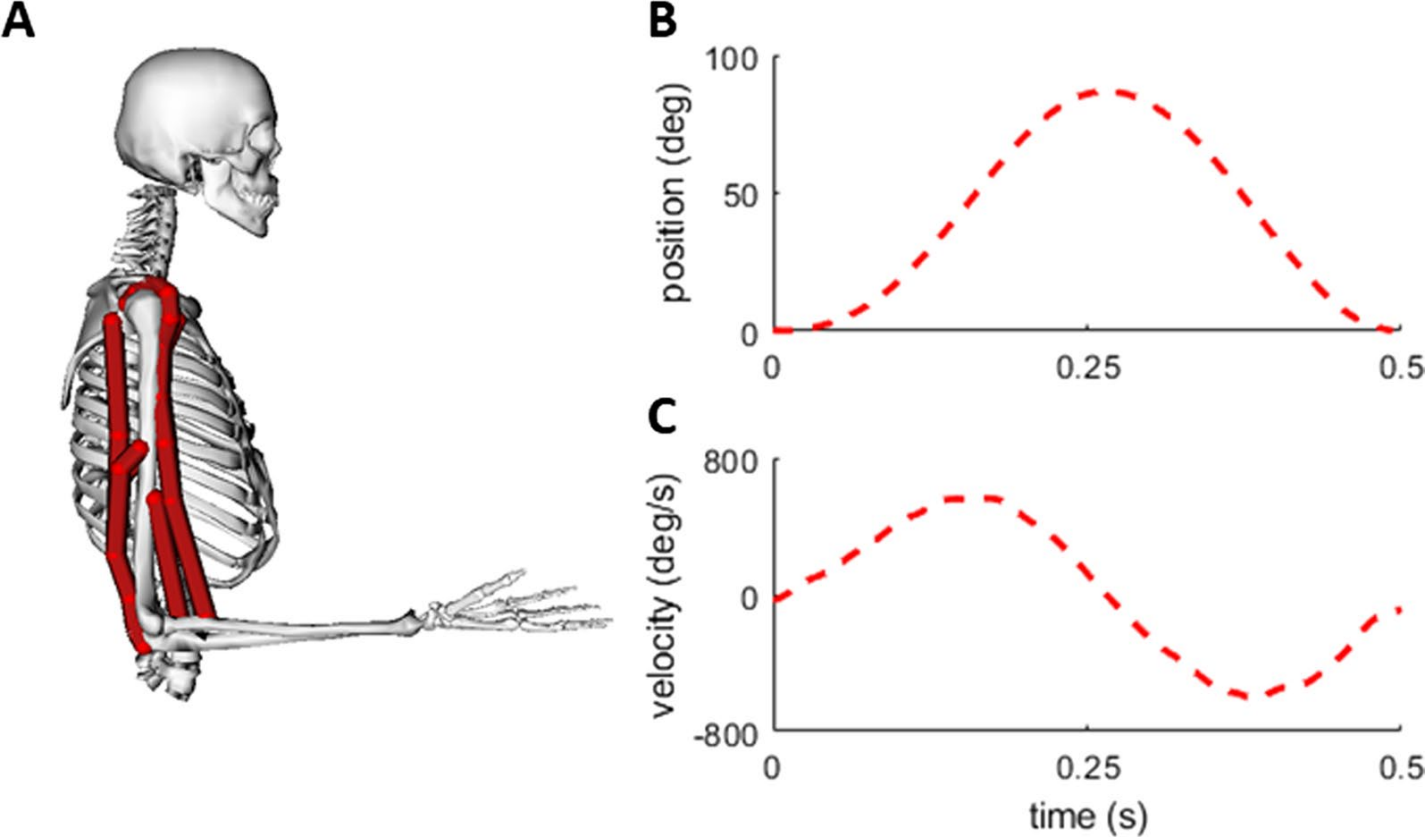
Elbow Musculoskeletal Model and Reference Data: **A** OpenSim elbow model with the elbow posed at 90 degrees (mid-point position). Red lines represent the paths for each of the six muscles in the model. The reference position (**B)** and velocity (**C)** trajectories used as input into the MCMC log likelihood function

We used an MCMC algorithm to identify a range of plausible muscle excitations that could produce a simulated elbow flexion–extension motion in a musculoskeletal model. We used a modified musculoskeletal model (arm26; adapted from: [22]) available on the OpenSim 3.3 software package (Fig. 1A; [9]). We modified the model such that it had only a single mechanical degree-of-freedom (DOF), representing flexion and extension of the elbow joint. The elbow was actuated by six muscle–tendon units (Millard2012EquilibriumMuscle; [43]): three elbow flexors (brachialis, biceps brachii long head, biceps brachii short head) and three elbow extensors (triceps brachii lateral, triceps brachii medius, triceps brachii longus). We also modified the muscle–tendon properties so that the series elastic elements for all six muscles were treated as rigid for computational speed.

We performed the following steps to create a “ground truth” reference motion to input into our MCMC analysis. First, we created a smooth elbow flexion–extension position trajectory that began at full elbow extension (0°), flexed to 90°, then extended back to full extension in a time of 0.5 s. We then used this flexion–extension motion as an input to OpenSim Moco to generate a set of “ground truth” muscle forces [44]. Briefly, OpenSim Moco is a software toolkit that allows users to solve a wide range of computational biomechanics problems. Here, we used OpenSim Moco to compute the optimal muscle excitations that would produce the created flexion–extension motion with our modified musculoskeletal model, by solving an optimal control problem that tracks the desired kinematic trajectory while minimizing the sum of the integrated muscle excitations cubed. The muscle force outputs from OpenSim Moco were used to compare with the MCMC analysis. Finally, we wanted to replicate the inherent noise in collecting kinematic data, so we added noise at each time point to the position data (noise = normal distribution, centered at 0, SD = 0.01 radians). We then performed a standard data processing workflow of smoothing the position data using a low-pass Butterworth filter with a cutoff frequency of 15 Hz, and then we differentiated the smoothed position data to compute velocity. The smoothed position trajectory and differentiated velocity trajectory were used as inputs into the MCMC analysis to define the elbow kinematics.

### Markov-chain monte carlo analysis

**Fig. 2:**
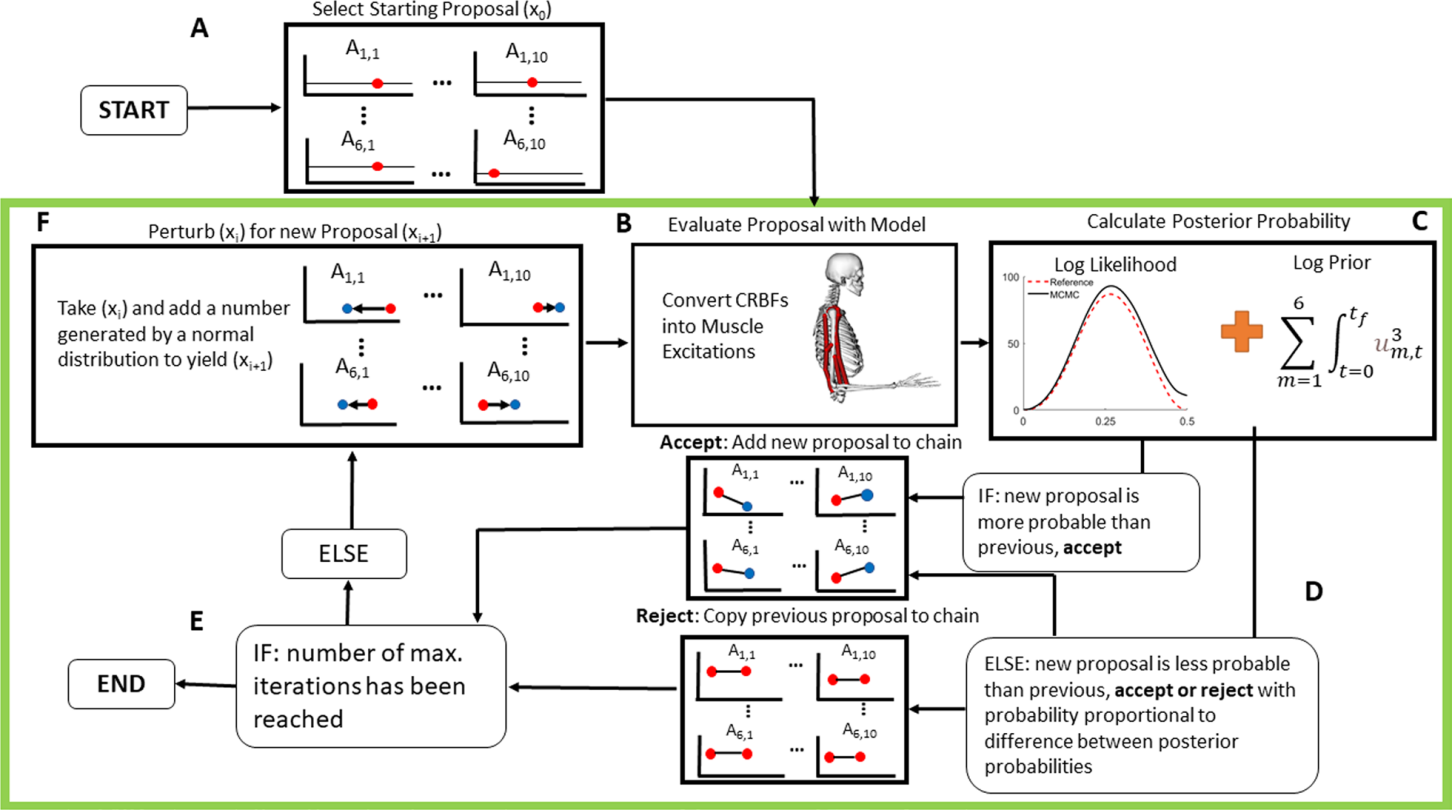
Flow Chart for MCMC and Elbow Flexion System: **A** The starting proposal for each parameter is drawn from a uniform distribution between [− 15,-5]. There are 60 parameters total representing amplitudes of the compact radial basis functions (CRBFs), 10 parameters for every muscle, where A_1,1_ is the amplitude of the first node of the first muscle, and A_6,10_ is the amplitude of the tenth node of the sixth muscle. **B** The proposal is converted from the set of CRBFs into a muscle excitations (Eqs. 1–2), which are given to OpenSim to generate a reference motion. **C** The posterior log-probability is calculated from the log likelihood (sum of square errors to the reference motion) and the log prior (the sum of muscle excitations (*u*) cubed). **D** The current proposal is accepted or rejected based on the change in posterior log probability from the original proposal to the new proposal (initial proposal is always accepted). **E** If the current iteration is equal to the pre-defined maximum iterations, the MCMC exits, otherwise it generates a new proposal in **F** by perturbing the current proposal by a value drawn from a normal distribution and continue to loop through the steps within the green box. Further details on the algorithm and acceptance criteria are given in [39, 45]

We used an MCMC algorithm based on a Delayed-Rejection, Adaptive Metropolis (DRAM) formulation [39, 45] to sample from the posterior distribution of parameters defining muscle excitations (Fig. 2). For this project, we defined the free parameters in the MCMC analysis to be transformed amplitudes of muscle excitations across time for each muscle. We generated the time-varying muscle excitation signals by defining a set of ten compact radial basis functions (CRBFs), summed over the time of the simulation for each muscle [46]. Each CRBF had a fixed center (c) and width (w), while the amplitudes (A) of each CRBF were the parameters in the MCMC algorithm. The centers of each CRBF were evenly spaced between t = -0.05 and t = 0.55 (intervals of 0.0667 s), and the widths were defined as two-times the distance between consecutive nodes (0.133 s). For each muscle, the ten CRBFs (F_m_) were then summed:

1 $${F}_{m}\left(t\right)={\sum }_{i=1}^{10}{A}_{i}{e}^{\left[1-\left(\frac{1}{1-{\left(\frac{t-{c}_{i}}{w}\right)}^{2}}\right)\right]}$$

**Fig. 3:**
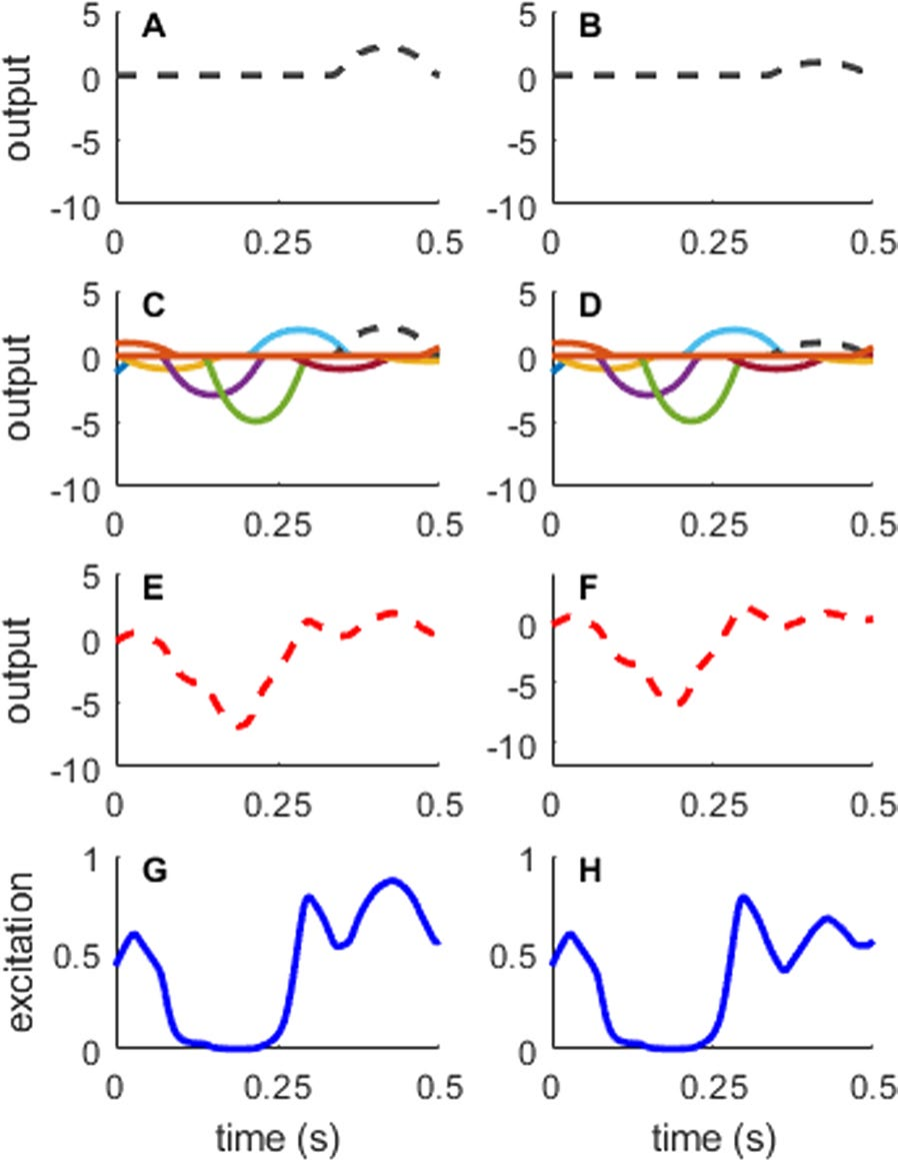
Generating muscle excitations from compact radial basis functions (CRBFs): The excitation signal for each muscle was determined by the amplitudes of ten CRBFs, each with a pre-defined center and width. **A** One CRBF shown individually. **C** All ten CRBFs with preset centers and widths, with varied amplitudes. **E** The sum across all ten CRBFs for a given muscle. **G** The summed output is then converted via an inverse-logit transform (Eq. 2) so that the excitation is constrained to be between 0 and 1. These excitations are then sent to the OpenSim model to perform the forward integration. The right column **B**, **D**, **F** and **H** shows the effect of changing the amplitude of a single CRBF (black dashed line). Changing the amplitude of the CRBF affects the muscle excitation signal within the width of the CRBF, but the area outside of the CRBF remains constant

*A*_*i*_ is the amplitude of the *i*th CRBF, and *t* is the time vector from 0 to final time (*t*_*f*_ = 0.5 s). The sum of the ten CRBFs (F_m_) were then transformed such that they ranged from 0 and 1 using an inverse logit-transform (Eq. 2; Fig. 3).

2 $${u}_{m}\left(t\right)=\left\{\begin{array}{c}\frac{1}{1+{e}^{\left({-F}_{m}\left(t\right)\right)}}if{F}_{m}\left(t\right)>0\\ \frac{{e}^{\left({F}_{m}\left(t\right)\right)}}{1+{e}^{\left({F}_{m}\left(t\right)\right)}}if{F}_{m}\left(t\right)\le 0\end{array}\right.$$

where u_m_(t) is the muscle excitation for the *m*th muscle. The inverse logit function was computed in a numerically stable way, which ensures the exponential function is only applied to negative arguments. Each *A*_*i*_ was constrained to be between − 30 and 30 (representing a uniform prior on individual coefficients), since values outside of these bounds, once converted with the logit transform, are approximately equal to 0 (for values less than -30) or 1 (for values greater than 30), therefore searching outside of these bounds becomes redundant.

The process for a standard Metropolis–Hastings MCMC algorithm begins by setting an initial, user-defined proposal for the amplitudes of the CRBFs, then simulates the model and compares the resulting output with the true data set. The initial proposals were determined for each of the sixty parameters, for each of the seven parallel runs that we performed, by generating a vector of random numbers on a uniform distribution between -15 and -5. The random initial proposal means that each MCMC run begins with an improbable proposal because the kinematics resulting from this random proposal will not match the reference kinematics and the sum of the integrated muscle excitations will be low. The parameters of the CRBFs define the set of muscle excitations (${u}_{m}\left(t\right)$) that are then sent to OpenSim to run a forward dynamics integration to solve for the corresponding kinematics. The forward integration uses inputs from the muscle excitation trajectories and the initial states of the model (initial elbow angle (0°), angular velocity (0°/s), and initial muscle activations (all muscles = 0.02)), and calculates the motion of the model through a forward integration of the set of muscle excitations to generate the kinematics for the current iteration of the MCMC algorithm. The forward dynamics integrations account for the muscle excitation-activation and force–length-velocity relationships of the contractile elements. For each proposal, the simulated kinematics of the current proposal were compared to the reference motion by computing the sum of squares error between the reference and position and velocity trajectories of the proposal to generate a log likelihood function (Eq. 3).

3 $$logLikelihood=\frac{-1}{2}\left[{\sum }_{t=0}^{{t}_{f}}{\left(\frac{{\theta }_{ref}\left(t\right)-{\theta }_{pro}\left(t\right)}{0.4}\right)}^{2}+{\left(\frac{{\dot{\theta }}_{ref}\left(t\right)-{\dot{\theta }}_{pro}\left(t\right)}{1.6}\right)}^{2}\right]$$

${\theta }_{ref}\left(t\right)$ is the reference angular trajectory, ${\theta }_{pro}\left(t\right)$ is the position trajectory of the current proposal, ${\dot{\theta }}_{ref}\left(t\right)$ is the reference angular velocity trajectory, and ${\dot{\theta }}_{pro}\left(t\right)$ is the velocity trajectory of the current proposal. The denominators in each term were heuristically chosen to give each term a relatively equal contribution to the log likelihood function on the basis that a-priori all errors are of approximately equal contribution, another option would be to define these as additional parameters with their own prior.

In addition to the log likelihood function, the posterior probability also included a log prior function to represent the physiological hypothesis that the central nervous system distributes muscle forces across agonist muscles in a way that reduces muscle fatigue [23, 42]. The log prior function was designed such that the sum of the integrated muscle excitations cubed in the MCMC were similar to that of the sum of the integrated muscle excitations cubed from the reference data (Eq. 4).

4 $$logPrior=\frac{-1}{2}\left[{\left(\frac{\left({\sum }_{m=1}^{6}{\int }_{t=0}^{{t}_{f}}{u}_{m,t}^{3}\right)}{0.08}\right)}^{2}\right]+U\left(A\right)$$

Here, *m* is an index corresponding to each of the 6 muscles, *t* is time and *u*_*m,t*_ is the muscle excitation of the *m*^*th*^ muscle and time *t*. The denominator for the log prior was selected to be arbitrarily wide, such that the search space was not overly constrained by the prior function definition. Based on our constraints of the amplitudes of the CRBFs between [− 30,30], the U(A) function is 0 on the allowed range [− 30,30] for each amplitude (A) value, and negative infinity outside that range. Therefore, U(A) allows any A value within those bounds, and rejects any A value greater than 30 or less than -30.

After the randomized, user-defined initial proposal was simulated, a new proposal is generated by adding noise to the initial proposal, then evaluating this new proposal with the model (Fig. 2). New proposals are always accepted if the log posterior probability density (Eq. 5) is more likely compared to the previous proposal.

5 $$logPosterior=logLikelihood+logPrior$$

The log posterior density describes a set of plausibility assumptions about how the motion occurred, but to make use of the information encoded in this mathematical model, we must have a way to compute with it. Fortunately, MCMC techniques provide us with a computationally convenient way to draw approximate samples from the probability distribution described by the log posterior density. MCMC finds each new proposal by assessing the new proposal relative to the preceding proposal. If the new proposal has decreased log probability density compared to the previous proposal, it is accepted or rejected with a probability based on the difference between the log probability density values of the two proposals (e.g., if the relative difference between the new proposal and the previous proposal is 0.8, then the new proposal is accepted with a probability of 80%, similarly if the relative difference between proposals is 0.1, then the new proposal is only accepted with a probability of 10%). If the new proposal is accepted, it becomes the next sample in the chain. If the current proposal was rejected, we utilized a Delayed-Rejection algorithm to resolve rejected proposals. After a rejection of a proposal, instead of immediately retaining the previous proposal and advancing to the next iteration, a second-stage proposal is evaluated [39]. The second-stage proposal is an intermediate point between the previous iteration in the chain and the rejected proposal (see [39] for mathematical details). If the second-stage proposal was also rejected, the algorithm copies the previous proposal from the previous iteration to the current iteration. Once this process is completed, we move to the next iteration of the algorithm, where a new proposal is generated and evaluated.

This process continues iteratively until a predetermined stopping point is reached, usually based on the total number of iterations (Fig. 2). Lastly, as the MCMC algorithm iterates, we used an Adaptive Metropolis algorithm to tune the proposal distribution using the history of the chain with the goal of obtaining reasonable results without excessive iterations [45]. The Delayed-Rejection and Adaptive Metropolis algorithms work together to improve the overall efficiency of the Metropolis–Hastings algorithm without requiring the calculation of derivatives with respect to parameter values as needed in some other algorithms based on Hamiltonian Monte Carlo, for example. Since the OpenSim software is a “black box” as far as the MATLAB-based sampler is concerned, it is important that the sampler does not rely on information other than the values of the log posterior density.

Seven parallel MCMC chains were run simultaneously (in parallel) starting from different initial proposals. The algorithm was set to terminate at 500,000 iterations, with the first 250,000 iterations discarded as “burn-in” (first 50%) before the results were evaluated. Since the initial proposals begin from a random state, these early proposals are unlikely to provide results that have typical posterior probability, therefore, a “burn-in” is used to allow the MCMC algorithm enough time to find areas in the search space that are of high likelihood.

### Performance analysis

We evaluated the performance of the MCMC algorithm based on (1) the total time for the algorithm to run, (2) whether the algorithm converged, (3) the root mean squared error (RMSE) between the MCMC positions and velocities compared with the positions and velocities of the reference trajectory, and (4) whether the resulting muscle forces from the MCMC were comparable to the forces from the reference data. The seven parallel MCMC simulations were evaluated for convergence by calculating and plotting the rank-transformed values of A across all chains, as a subjective means of assessing convergence [47]. When converged, these rank plots should show a uniform distribution between 0 and 1 in all chains. This indicates that each MCMC simulation is exploring the same space as the whole ensemble. If the rank plot is flat across the range, then we have evidence that the MCMC algorithm has converged. In addition, we also evaluated the convergence diagnostics for the potential scale reduction statistic ($\widehat{R}$) and effective sample size for each of the sixty parameters [48, 49]. $\widehat{R}$ gives an indication of how well the parallel chains have mixed and reached equilibrium, with values less than 1.10 commonly accepted as converged. The effective sample size gives an estimate of the number of independent samples in the chain, since the MCMC chains will typically have some level of autocorrelation within a chain. Typically, an effective sample size of about 30 samples will allow for a reliable calculation of the means and standard deviations of the results according to the central limit theorem though a greater sample size is generally better.

We also computed correlation coefficients between pairs of parameters to understand how parameters correlated across time (correlating amplitudes at each time node) or across muscles (correlating amplitudes for each muscle). We expected the correlation analysis to demonstrate the relationships inherent in the muscle redundancy problem. Therefore, we predicted that the forces generated by agonist muscles should have negative correlations with each other at a given time point (i.e., as one muscle increases force, an agonist muscle would decrease force to match the torque requirements). Additionally, if there were antagonist co-activations in the results, we would see positive correlations between antagonist muscles at a given time point (i.e., as one muscle increases force, an antagonist muscle would increase force to result in co-activations). We also predicted that there would be negative correlations within a muscle between adjacent time points (e.g., nodes 1 and 2), since these parameters determine the excitation amplitude for overlapping regions of time (see Fig. 3C, D), while correlations between distant time points (e.g., nodes 1 and 10) to have only weak correlations as these points represent vastly different aspects of the movement. For example, a similar activation level for a given period of time could be achieved with a relatively greater amplitude in node 1 in exchange for a relatively lesser amplitude in node 2, whereas we predict that the amplitude in node 1 will have little to do with the amplitude in node 10.

We compared the forces from the MCMC results to the force trajectories of each muscle from the reference motion. The forces from the reference motion were computed directly with OpenSim based on the muscle excitations used to drive the motion of the model to produce the reference position and velocity trajectories. For this analysis, we sampled from 25 evenly spaced proposals (one every 10,000 iterations after the burn-in phase) from each of the seven parallel chains (175 total proposals) and calculated the means and standard deviations of the positions, velocities, and muscle forces of the samples. To assess if the MCMC algorithm tracked the reference kinematics, we computed the RMSE between the kinematics of the samples and the reference motion. The MCMC algorithm was performed on an eight-core desktop computer with a 2.1 GHz Xeon-Silver 4110 processor and 32 GB RAM.

## Results

**Fig. 4:**
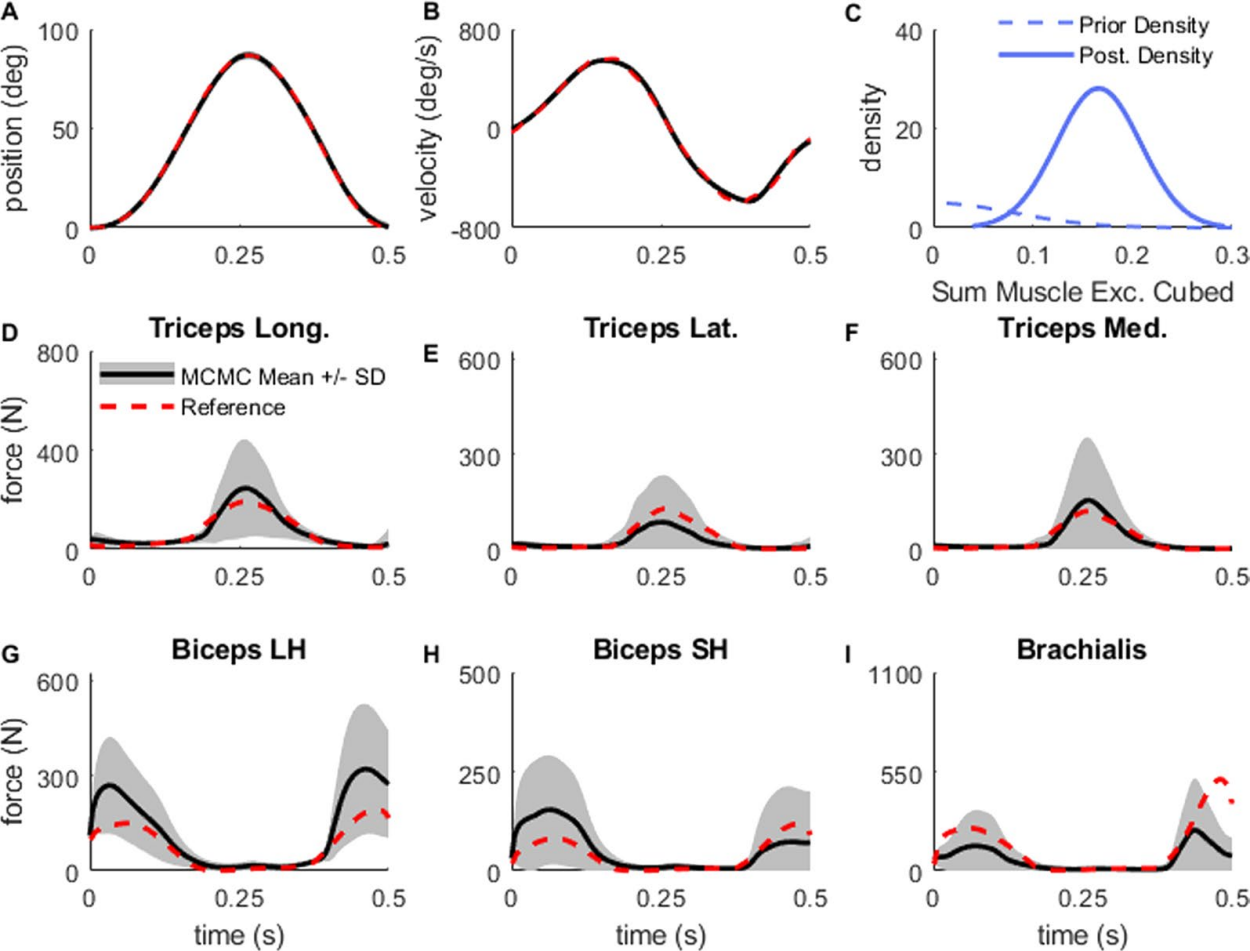
MCMC Results and Analysis: The position (**A)** and velocity (**B)** trajectories matched closely with the reference (red dashed line). **C** The prior (blue dashed) and posterior (post.) density (blue solid) on sum of muscle excitations cubed. The mean (black solid line) and 1 standard deviation (gray shaded region) of muscle force trajectories for triceps long head (**D**), triceps lateralis (**E**), triceps medialis (**F**), biceps long head (**G**), biceps short head (**H**), and brachialis (**I)** compared with the forces from the reference trajectory (red). For each of the muscle force subplot, the maximum value on the y-axis represents the peak isometric muscle force of the muscle

It took 55 h for the MCMC algorithm to perform 500,000 iterations for each of the seven parallel chains. The sampled trajectories tightly tracked the position (Average RMSE = 2°; Fig. 4A) and velocity data (Average RMSE = 32°/s; Fig. 4B). The sum of the integrated muscle excitations cubed in the posterior were approximately normally distributed as 0.16 ± 0.06 (Fig. 4C). When evaluating the range of plausible muscle forces for each of the six muscles in the model, the three elbow flexors produced force at the beginning of the motion (Fig. 4G–I) to accelerate the elbow into flexion. Then, the three elbow extensors decelerated the elbow during the mid-point of the motion (Fig. 4D–F). Lastly, the elbow flexors muscles again acted at the end of the motion to move the elbow to its final position.

When evaluating the sampled force results against the forces that produced the reference motion, the average elbow extensor and average elbow flexor forces from MCMC matched extremely close with the reference. One notable deviation between the average MCMC output and the reference forces occurred at the end of the motion where the brachialis in the average MCMC result produced slightly less force than the reference data, but the biceps long head produced slightly more force in the MCMC than the reference. Overall, there was no evidence of any strong co-activation between agonist and antagonist muscles.

**Fig. 5:**
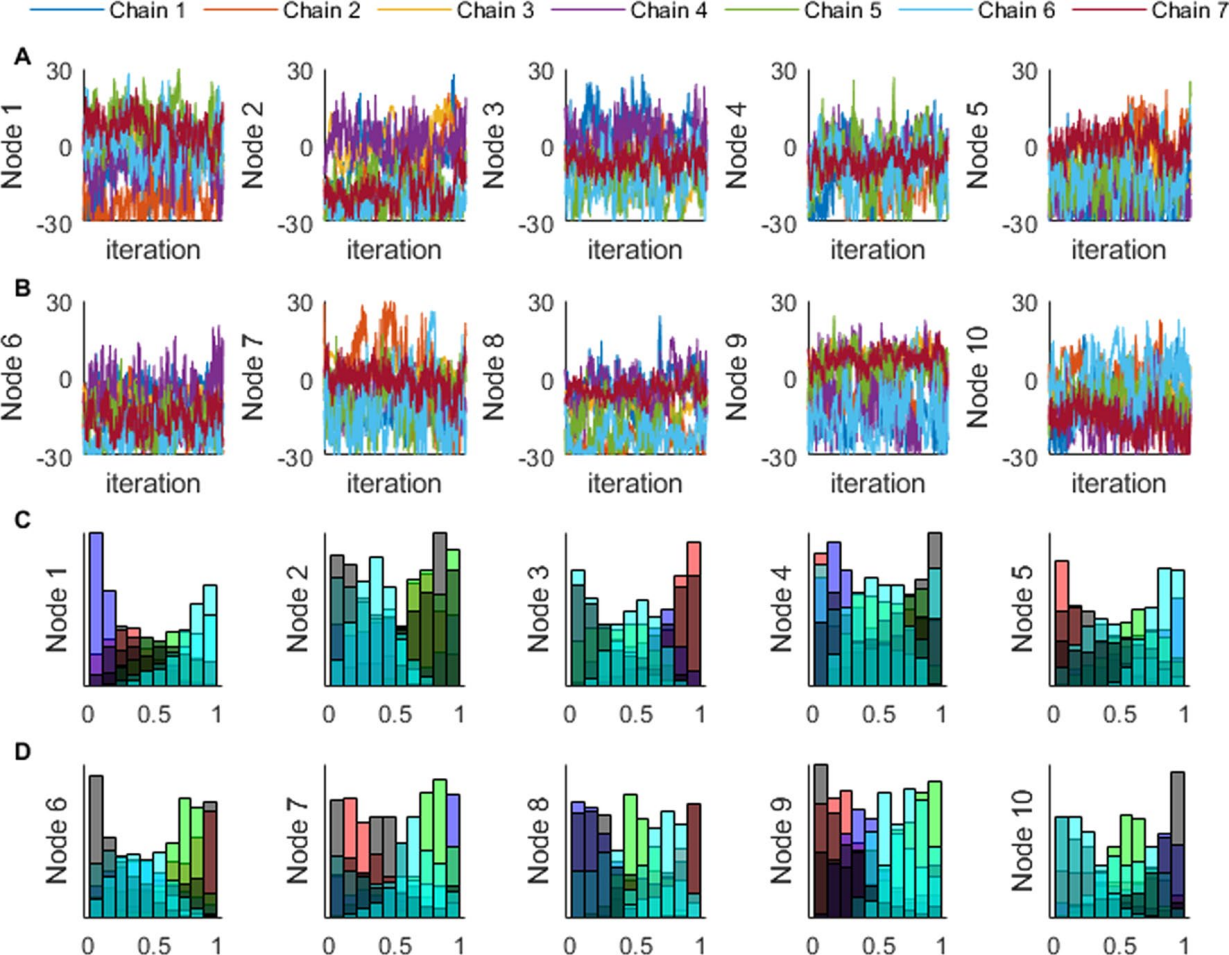
Chain and Rank Plots for the Brachialis: Chains (rows **A **and **B**) and rank plots (rows **C** and **D**) for each of the ten nodes for a representative muscle. Overall, the rank plots show that the seven chains did not fully mix, although nodes 5 and 8 have better mixing than nodes 1 and 10

**Fig. 6:**
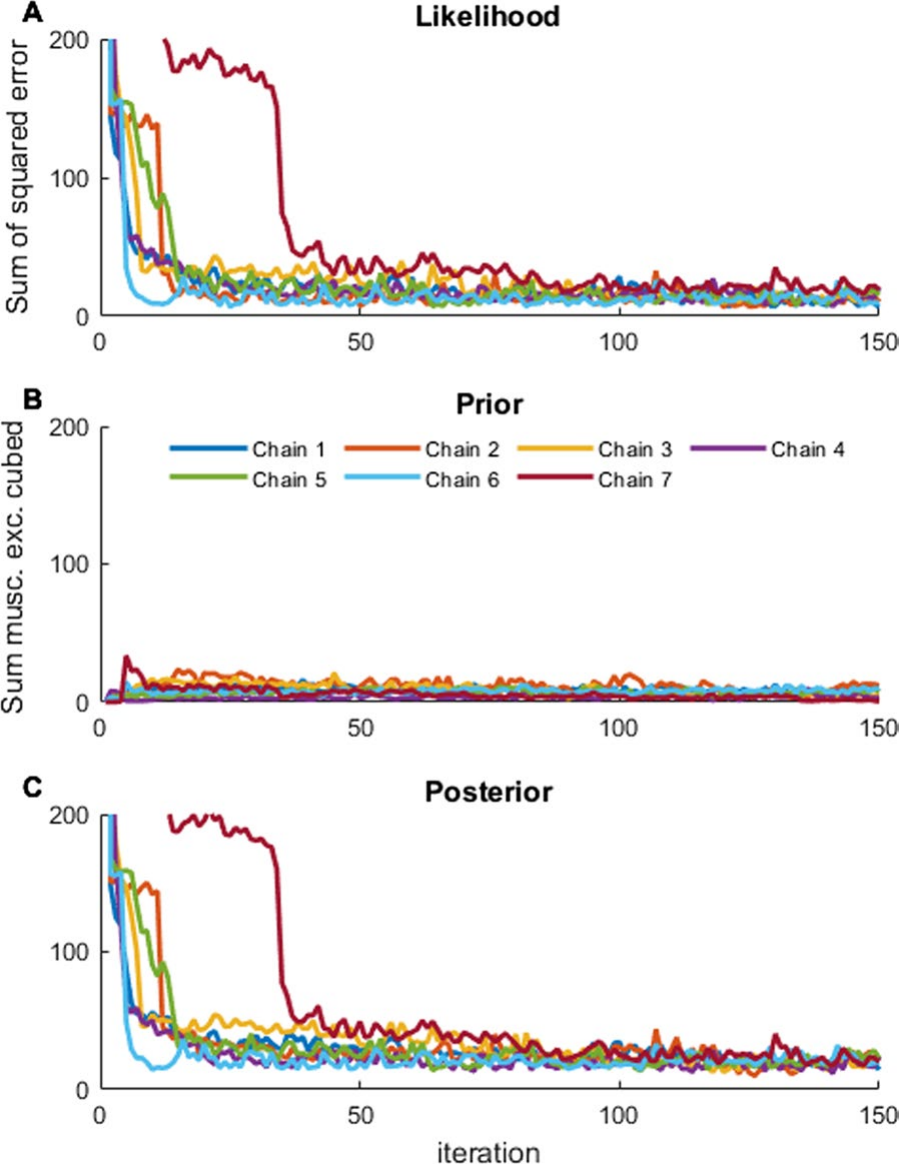
Likelihood, prior, and posterior for the first 150,000 iterations: This figure demonstrates that each of the seven parallel chains reach an equilibrium point in their output by the end of the 150,000th iteration, during the burn-in phase of the MCMC analysis. The raw output for the likelihood function shows a rapid decrease in sum of squared error within the first 50,000 iterations for each chain, eventually reaching an equilibrium point (**A**). The sum of integrated muscle excitations (Prior) has some early peaks during the MCMC chain, but also reaches equilibrium by 150,000 iterations (**B**). Finally, the sum of the likelihood and prior gives the posterior output (**C**). Note that the MCMC algorithm continues after the end of the plotted data to reach 500,000 iterations total

Overall, our rank plot analysis showed that the seven chains were not fully mixed after the MCMC analysis terminated. In some cases, the chains might have become stuck in local regions (brachialis; Fig. 5A, B). The rank plots for each of the parameters did not have a flat distribution, indicating the parallel chains were not searching the same solution spaces (brachialis; Fig. 5C, D). Across all sixty parameters, the $\widehat{R}$ statistic ranged between 1.05 to 2.64, with two of sixty parameters below the tolerance threshold of 1.10. The effective sample size ranged from 32 to 105 independent samples across all parameters (representative muscle: brachialis; Table 1). While the MCMC convergence diagnostic tests that we computed did not meet standard thresholds, our posterior likelihood output shows that even before the end of the burn-in phase, the overall outputs had reached an equilibrium point (Fig. 6). Overall, this provides evidence for the high correlations among parameters in musculoskeletal modeling.

**Table 1:** Convergence diagnostics for a representative muscle (brachialis): the potential scale reduction statistic ($\widehat{R}$) where values < 1.10 are considered to indicate that the chains are in equilibrium, and the effective sample size indicating the number of independent samples in the chain

Parameter	R-hat	Effective sample size
Brachialis Node 1	1.69	36
Brachialis Node 2	1.44	37
Brachialis Node 3	1.40	52
Brachialis Node 4	1.05	35
Brachialis Node 5	1.12	60
Brachialis Node 6	1.17	69
Brachialis Node 7	1.25	55
Brachialis Node 8	1.86	79
Brachialis Node 9	1.66	73
Brachialis Node 10	1.60	50

**Fig. 7:**
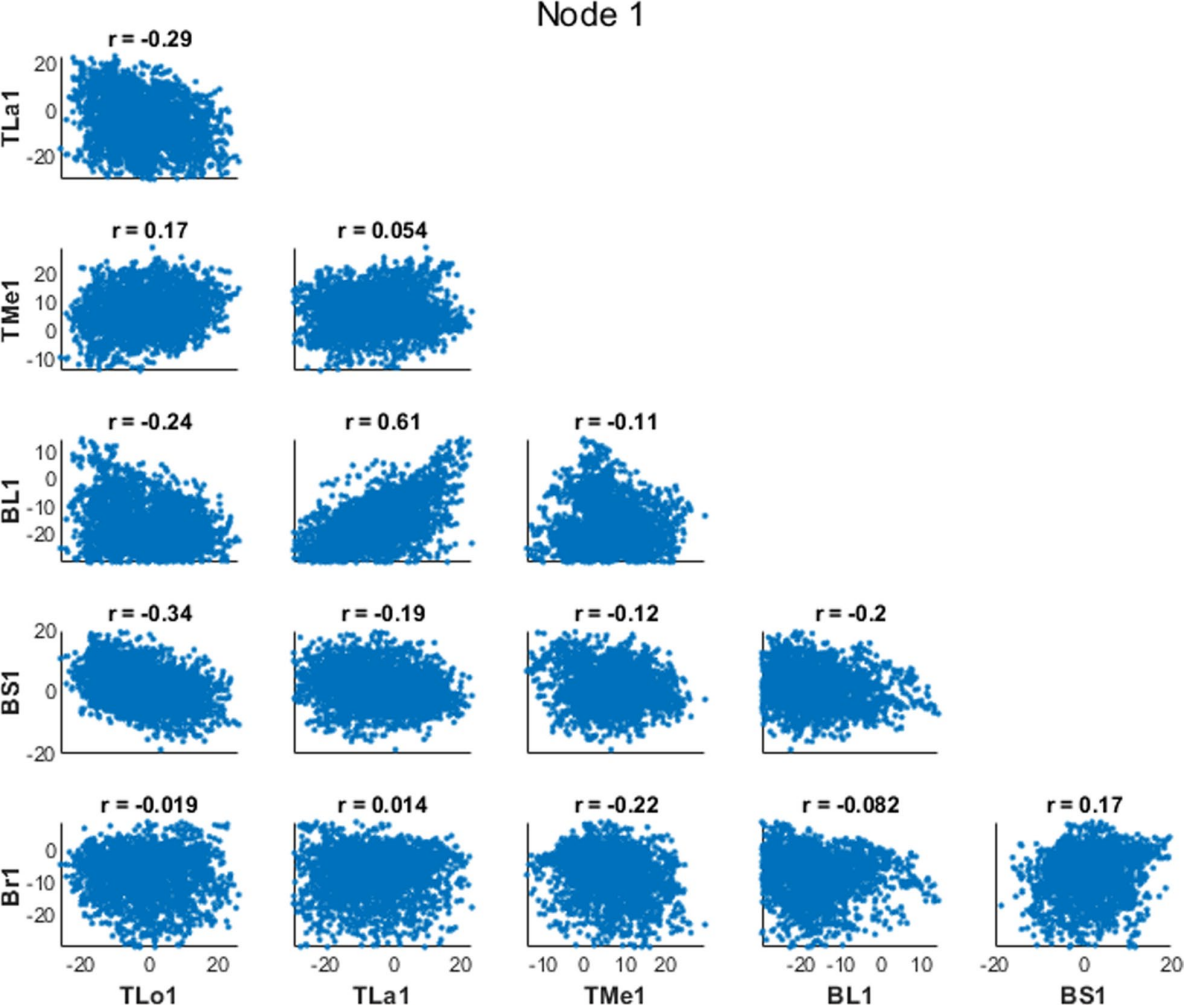
Node 1 Correlations: Correlation plot between the six muscles in the model for the parameters representing the first time point, or node: triceps long head (TLo1), triceps lateralis (TLa), triceps medialis (TMe1), biceps long head (BL1), biceps short head (BS1), and brachialis (Br1)

**Fig. 8:**
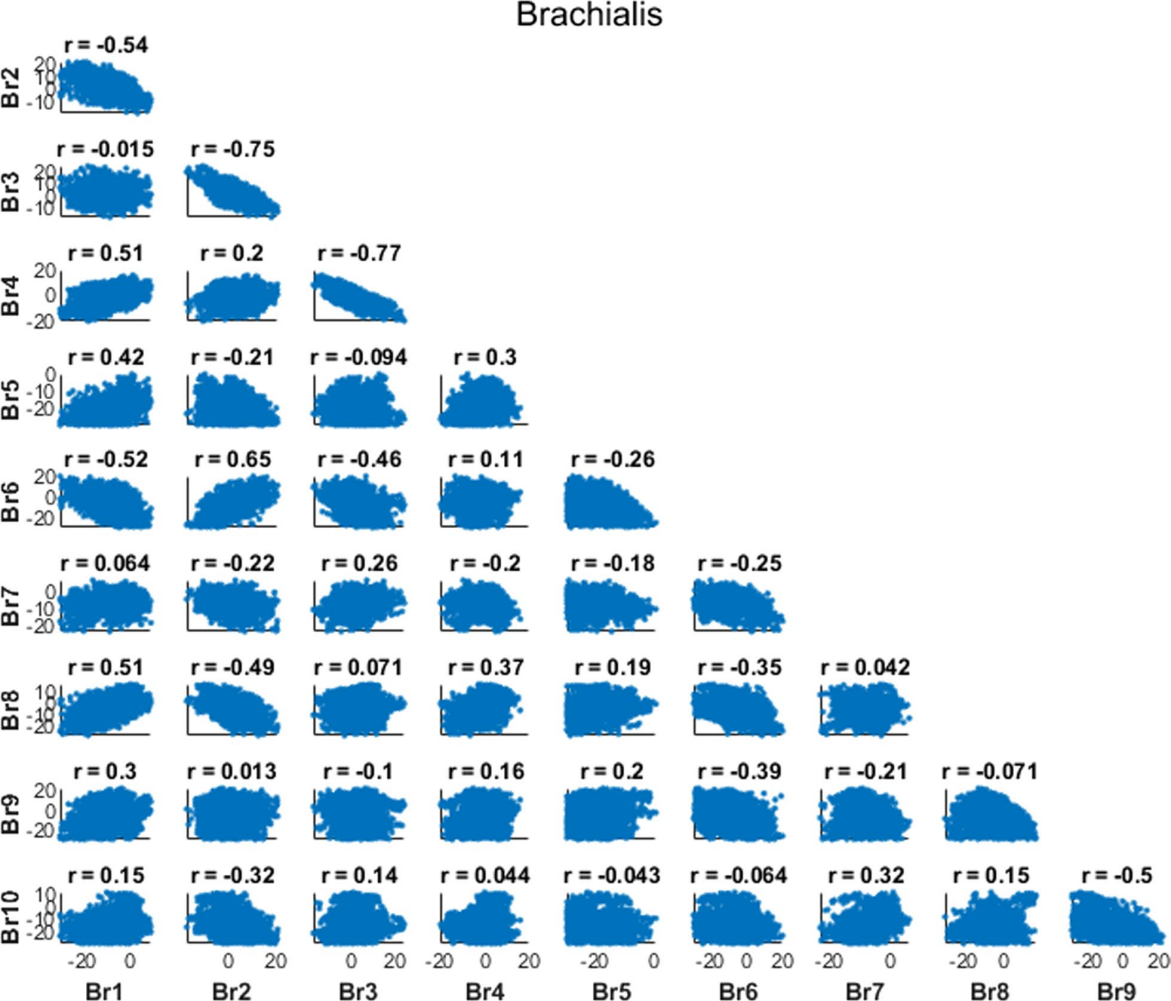
Brachialis Correlations: Correlation plot between the ten nodes of the brachialis (Br) muscle, where Br1 is the amplitude of the parameter for the brachialis at Node 1, and Br10 is the parameter for the brachialis at Node 10

There were significant correlations between pairs of coefficients within a node, however many of the correlations had a weak r value (Fig. 7: between muscles for the time node 1). One correlation between the biceps long head and the triceps lateralis reveal co-activation strategies explored by the algorithm (r = 0.61). This correlation shows that when the excitation level is greater for the triceps lateralis during initial elbow flexion, the biceps long head excitation must be greater as well to match the kinematics of the movement at the time. There were stronger relationships when we investigated correlations between nodes within a muscle, like for the brachialis (Fig. 8). Generally, correlations between adjacent time point nodes (the plots along the diagonal of Fig. 8) had negative correlations, as we had predicted. Many of the other correlations among non-adjacent time nodes had weaker correlations.

## Discussion

A key question in motor control and biomechanics is how our nervous system controls our muscles during movements, and therefore how muscle forces are distributed across muscles [8, 17, 23]. Musculoskeletal modeling and simulation present a way to estimate the muscle forces during a given motion, but these methods rely on several modeling assumptions. Standard methods for estimating muscle forces result in estimates of a single time-series of muscle forces for a given movement, which disguises the real uncertainty that accompanies the unknown model parameters and other related assumptions used for musculoskeletal modeling studies. In this paper, we presented a workflow and evaluated the feasibility of using Bayesian estimation and MCMC to find a plausible range of muscle forces that could result in a simulated elbow flexion–extension task, while potentially allowing for any mathematical hypothesis about the motor control problem. In order to define the potential solution space, we used a prior probability distribution intended to represent physiological evidence that muscle forces are distributed across agonist muscles in a way the reduces muscular effort [23]. This approach resulted in a range of muscle forces that reproduced reference kinematics and generally matched with muscle forces from the reference trajectory (i.e., the ground truth), while illustrating the array of plausible combinations of muscle forces that could give rise to the reference elbow motion.

In this paper we have demonstrated two important techniques. The first is the quantification of uncertainty through a Bayesian model of our understanding of the mechanical process, this model is defined by a high dimensional function of the parameters called the log posterior probability density. The second is the computational technique of sampling from that model using MCMC to make statements about which behaviors are more or less plausible. The Bayesian modeling technique is a very general-purpose way to describe all of the potential uncertainties that are involved in a mathematical model. MCMC computation is a general-purpose way to sample from probability distributions such as the ones created in a Bayesian model. Although our convergence diagnostics showed that the particular algorithm employed in the MATLAB library was not able to sample effectively from the entirety of the plausible region of parameter space, all chains did converge into the high probability set and they all represent plausible samples from the possible motions. The lack of complete convergence in all chains shows only that more advanced MCMC algorithms should be investigated which can better sample from the complex high dimensional posterior distributions inherently created by these dynamic models in order to more completely characterize the mathematical model described by the log probability density.

A key result from this paper is that Bayesian inference effectively quantifies different plausible combinations of muscle force trajectories, with comparable total muscle effort, that can give rise to very similar kinematics. An important consideration for the computed range of plausible muscle forces in this problem is a user-defined choice for the denominator of the prior probability function, which represents the expected variance in the sum of the integrated muscle excitations cubed. We chose to parameterize the prior function with a center around 0 and an arbitrarily wide variance, however other users may choose to specify different parameters based on their prior beliefs about the motion being performed. For example, creating a narrower prior probability density, which would indicate a high degree of confidence in the hypothesis of minimizing muscle effort, would allow for a narrower region of plausible muscle forces in our results (Fig. 4). Instead, a wider prior probability density might be appropriate for clinical populations where large co-activations among antagonist muscles may be expected.

Our Bayesian approach allowed for uncertainty in the kinematic data by using a likelihood function that represented error in the positions and velocities from the reference trajectory. Other implementations could allow for greater uncertainty in these trajectories by increasing the denominator in each of the terms of the likelihood function (Eq. 3), especially if there was a large uncertainty in the collected kinematic data or by using these values as additional parameters with their own prior uncertainty. For example, if kinematic data were collected via retroreflective markers with greater likelihood for measurement error, due to skin or clothing movement relative to bony landmarks, the MCMC implementation could set wider distributions for those variables to capture the uncertainty in the data.

Our approach builds upon previous work by utilizing a prior probability function, which is based on the physiological hypothesis of minimization of muscle effort, to constrain the solution space and provide more plausible bounds on minimum and maximum forces during the movement. Representing the hypothesis of minimization of muscle effort with a prior probability function allows for a balance between the singular solution from standard optimization routines and the relatively unbounded solutions from previous work [31–33]. Computing the sum of the cubed muscle excitations integrated over time is one hypothesis for how the nervous system deals with the muscle redundancy problem during motor control, and it is a common objective function used in many musculoskeletal modeling optimizations [8]. Related work has sought to balance the reproduction of measured kinematic data while estimating muscle forces, such as estimating kinematics and muscle forces during downhill skiing, where the collected kinematic data can be very noisy [50]. Additionally, other work has used IMU data collected on body segments, which can be difficult to resolve to traditional kinematic joint angle data, to estimate kinematics and muscle forces [51]. However, each of these approaches still results in a single optimal trajectory output, but future iterations could implement a Bayesian model with values computed by MCMC to better capture the uncertainty in noisy or unreliable data.

One advantage of using the prior probability function is that it can allow the user to change the parameters of the function to specify a broad set of plausible motor control hypotheses, thereby modifying the range of the plausible muscle forces. While our study focuses on the uncertainty in kinematic data and model of motor control (i.e., the objective function), a potential advantage of this Bayesian approach is the ability to include many different sources of data or other hypotheses within the prior density function [52, 53], or include other types of uncertainty such as model parameter estimation or different types of measurement error [18–21, 28–30]. For example, future implementations of this paradigm could utilize EMG data for some muscles, while still constraining the solution space for muscles without EMG data (expanding on [31]).

Additionally, a Bayesian inference approach could also be combined with EMG-driven optimizations [e.g.,^54^] to represent and quantify the uncertainty in these optimizations. EMG-driven optimization methods seek to find a set of physiologically based parameters for a musculoskeletal model that, given a measured EMG signal, would result in the corresponding measured kinematics. With this method, uncertainty can arise from the noise in EMG signals, normalizing EMG data to a maximum activation, the measured kinematics, or other musculoskeletal model parameters. Using a Bayesian inference approach to represent the uncertainty in the EMG or kinematic data or the muscle parameters would help researchers quantify a range of plausible forces that could result from the data set.

We expected our correlation analysis to reveal clear demonstrations about how the nervous system can solve the muscle redundancy problem, with negative correlations between agonist muscles and positive correlations between antagonist muscles at a given time point. We further expected that correlations within muscles across time points would reveal negative correlations between adjacent nodes and weaker correlations between distant nodes. While we did reveal some strong correlations among the parameters that followed our predictions, some of the correlations revealed unexpected relationships between parameters (e.g., brachialis node 2 and brachialis node 6), suggesting that there may be non-intuitive relationships in how the muscle redundancy problem gets solved for a given motion. This is yet another advantage of the Bayesian approach, revealing potentially non-intuitive correlations between parameters in a system.

Other previous work has used sensitivity analyses for musculoskeletal modeling studies by systematically varying the objective function and re-solving the optimization problem for each function [17, 25–27] or searching through a range of potential unknown modeling parameters [18, 28–30]. While these approaches can allow for an exploration of the sensitivity of modeling assumptions to the overall results, there can be important limitations to traditional sensitivity analyses. Typical sensitivity analyses vary model parameters at random based on known or predicted measurement error for a set of parameters of interest (e.g., muscle insertion coordinates, or tendon slack lengths). The limitation with this approach is that it could end up evaluating sets of parameters that are highly unlikely to produce the measured data. A Bayesian inference approach differs from these traditional sensitivity analyses as the MCMC algorithm will only search through spaces where parameter values give rise to reasonable predictions of the data. For example, the MCMC algorithm will reject proposals with extremely low probability and often accept proposals with high probability. Therefore, the computational time spent in regions of low probability is minimized, while the algorithm explores the regions of high probability. This can dramatically improve computational efficiency when exploring complex search spaces.

One potential obstacle in implementing MCMC with time-varying signals is that the problem has the potential to be extremely high-dimensional. For example, if each hundredth of a second was represented with an amplitude for the 0.5 s simulation, we would have to search 300 total parameters (50 nodes times six muscles). We were able to reduce the dimensionality of the problem by using CRBFs to compose the muscle excitation signals used in our forward simulations. CRBFs are useful for generating time-varying signals for MCMC problems as they allow complex time-series trajectories to be represented with a few parameters [46]. CRBFs also have an advantage over other potential basis functions such as Chebyshev polynomials or Fourier series because they decouple the parameters from each other. For example, in a Chebyshev polynomial series, changing the value of just one coefficient will change the entire time-series trajectory, sometimes in dramatic ways (see Additional File 1; Figure A1). Instead, the parameters (representing amplitudes) of the CRBFs change the shape of the time-series trajectory only within the preset width around the center of the individual function (Fig. 4). Other equations for representing complex trajectories, such as B-Splines, could be implemented instead of CRBFs.

## Limitations

While this approach shows promise by producing muscle forces that compare favorably with the ground truth without being bound to singular solutions, there are still improvements that need to be made. Our rank plot analysis and convergence diagnostics ($\widehat{R}$) show that our MCMC chains were not converged, indicating that each of the parallel chains were not searching the same solution space throughout the MCMC. In MCMC, convergence is defined based on the scope of the total solution space observed. So not getting complete convergence doesn’t mean that the outputs we found are invalid, it simply indicates that each of our seven chains did not explore the entire solution space. The lack of convergence is a limiting factor to our methodology now, and one that is bound by current computational speed and MCMC algorithm efficiency, given an MCMC algorithm that can work for problems that do not have easily computed gradients. To verify that the algorithm we chose would converge when used for simpler mechanical models, we used it to successfully recover model parameters of a mass-spring-damper system (see Additional File 1). Improvements in algorithmic efficiency are critical to make this approach feasible for musculoskeletal modeling studies, especially studies that use more complex musculoskeletal models (e.g., gait models). For tasks like gait or other motions involving balance, this MCMC approach can still work well, as users can construct likelihood functions that would reject any motion that resulted in falling. However, the number of MCMC iterations would likely need to dramatically increase from what we did in this study due to the increase in model complexity. It may be possible to offset the time the algorithm spends searching infeasible solutions by providing initial proposals which already satisfy stable walking patterns, perhaps by using an optimization procedure to find a set of muscle excitations that can produce a desired gait pattern.

Future work in this area requires developing computational techniques able to search high-dimensional spaces in an efficient manner. Once greater algorithmic efficiency is achieved, more complex musculoskeletal models could be included in this type of analysis (e.g., gait models) and more parameters could be included (e.g., muscle model parameters such as peak isometric muscle force or tendon slack length). An alternative approach could include using differentiable musculoskeletal models or using algorithmic differentiation for OpenSim models [55], but each of these approaches also requires considerable computational set up time and expertise, which creates issues for broad applicability and reproducibility. Using a differentiable musculoskeletal model would allow for some different choices in MCMC sampling algorithms, such as algorithms that can utilize the slope of the solution space to better identify efficient ways to move with each iteration, and therefore converge in a faster amount of time.

## Conclusion

Altogether, this paper illustrates the type of analyses we could achieve with a Bayesian inference method applied to musculoskeletal modeling and simulation, allowing for the estimation of plausible muscle forces that could have generated an observed set of kinematics. The output from this analysis could then be applied to other outcome measures relevant to biomechanical modeling, such as providing a range of plausible values for metabolic energy or joint contact forces in musculoskeletal models for a given motion. This approach can allow us to better quantify the uncertainty that is characteristic to musculoskeletal modeling and simulation studies. Improvements to the algorithmic efficiency will make this method more feasible to merge into a new standard of musculoskeletal modeling and simulation. Therefore, further developments for MCMC search algorithms which specifically are usable for common musculoskeletal simulation tools (or other problems without easy access to model derivatives) should be at the forefront of future research, since these kinds of mechanics problems are difficult to sample from using the relatively simple techniques in the MATLAB MCMC package. Doing so will allow researchers to better represent uncertainty in musculoskeletal modeling and simulation studies in the future.

## Supplementary Information

**Additional file 1.** The additional file contains two sections: 1) Evaluating the feasibility of using Chebyshev polynomials with musculoskeletal models and MCMC. 2) Evaluating the MCMC algorithm with a simple mass-spring-damper model to recover mechanical parameters.

## Data Availability

The dataset supporting the conclusions of this article is available in the GitHub repository, https://github.com/russelljohnson95/MSM_Modeling_and_MCMC.
